# HPV-Related Cancer Prevention and Control Programs at Community-Based HIV/AIDS Service Organizations: Implications for Future Engagement

**DOI:** 10.3389/fonc.2018.00422

**Published:** 2018-10-26

**Authors:** Lisa T. Wigfall, Shalanda A. Bynum, Heather M. Brandt, Neethu Sebastian, Marcia G. Ory

**Affiliations:** ^1^Division of Health Education, Department of Health and Kinesiology, College of Education and Human Development, Texas A&M University, College Station, TX, United States; ^2^Department of Health and Kinesiology, Transdisciplinary Center for Health Equity Research, Texas A&M University, College Station, TX, United States; ^3^Center for Scientific Review, National Institutes of Health, Bethesda, MD, United States; ^4^Arnold School of Public Health, University of South Carolina, Columbia, SC, United States; ^5^School of Public Health, Texas A&M University, College Station, TX, United States; ^6^Center for Population Health and Aging, Texas A&M University, College Station, TX, United States

**Keywords:** community-based HIV/AIDS service organizations (ASOs), human papillomavirus (HPV) vaccination, men who have sex with men (MSM), people living with HIV (PLWH), vulnerable populations

## Abstract

**Background:** People living with human immunodeficiency virus/acquired immunodeficiency syndrome (HIV/AIDS) and, men who have sex with men (MSM) are disproportionately affected by genital warts and cancers caused by human papillomavirus (HPV). We assessed community-based HIV/AIDS service organizations' (ASOs) staff awareness, knowledge, attitudes, and beliefs about HPV and effective cancer prevention tools, namely HPV vaccination, Pap, and HPV tests. The potential engagement of ASO staff in future efforts to reduce the disproportionate burden of genital warts and HPV-related cancers among HIV-positive women and MSM was explored.

**Methods:** In May-June 2016, staff were recruited from three ASOs located in the South United States Census region—a geographical area disproportionately affected by HIV/AIDS. Participants completed a 30-min self-administered, 118-item paper and pencil survey about HPV and cancer. Data analysis was conducted using Stata/SE 14.2.

**Results:** ASO staff (*n* = 30) were 83% non-Hispanic Black, 40% lesbian/gay, and worked with people living with HIV for an average of 11.4 ± 7.7 years. All reported hearing of HPV and 77% had heard of the HPV vaccine (*n* = 23). While all knew HPV can cause cervical cancer, only 67% knew HPV can cause anal cancer. Most (61%) thought the HPV vaccine could prevent cervical cancer. Fewer (39–48%) thought the HPV vaccine could prevent anal, oral, penile, vaginal, and vulvar cancers. All were willing to encourage MSM and female clients to talk to a healthcare provider about HPV vaccination. Almost all were willing to promote HPV vaccination to clients (91–95%) and navigate clients to adult safety net HPV vaccine providers (86–95%). More than half (59–67%) thought they could positively influence their MSM and female clients' HPV vaccine decision-making.

**Conclusion:** HPV vaccination and the Pap and HPV tests are effective cancer prevention tools that can reduce the disproportionate burden of genital warts and HPV-related cancers among HIV-positive women and MSM. Engaging ASO staff in cancer prevention efforts may increase HPV vaccination rates and early detection of HPV-related cancers among HIV-positive women and MSM. Exploring ASOs as community-based settings for promoting effective cancer prevention tools may foster opportunities to reduce the disproportionate burden of genital warts and HPV-related cancers among HIV-positive women and MSM.

## Introduction

Human papillomavirus (HPV) is the most common sexually transmitted infection worldwide (1, 2). An HPV infection is usually transient and will clear within 1–2 years. In contrast, a persistent HPV infection can cause genital warts and some types of cancer (3). Some populations are disproportionately affected by genital warts and HPV-related cancers. HIV-positive women are three times more likely than HIV-negative women to develop cervical cancer (4). People living with HIV (PLWH) and MSM are disproportionately affected by anal cancer (4). MSM represent ~75% of HIV-positive males (5). MSM are disproportionately affected by anal cancer, and HIV-positive MSM bear an even greater burden of anal cancer compared to HIV-negative MSM.

Because of their increased risk for developing cervical cancer, HIV-positive women are screened more regularly than HIV-negative women (at least until a history of three consecutive normal Pap test results has been established) (6). Even with a normal Pap test history and negative HPV test result, co-testing intervals are every three years for HIV-positive women 30+ years old compared to every five years for HIV-negative women (6). Further, unlike cervical cancer for which screening tests like the Pap test and HPV DNA test are recommended, there are no recommended screening guidelines for anal cancer. Despite the absence of screening guidelines for anal cancer, some HIV-positive women, and men undergo anal Pap test screening because of their increased cancer risk (7–10).

Data from HPV vaccine clinical trials supports the benefits of HPV vaccination among at-risk populations disproportionately affected by HPV-related diseases and cancers, such as HIV-positive women and MSM, in light of minimal risks (e.g., adverse events such as syncope) and decreased effectiveness for those already exposed to some HPV types. Yet, HPV vaccine coverage remains suboptimal among MSM (11, 12), and little is known about HPV vaccine uptake among HIV-positive women. This is a missed opportunity for prevention given that the HPV vaccine and Pap tests have been effective cancer prevention tools. HPV-related diseases and cancers are preventable health conditions that negatively affect the quality of life of the individual diagnosed as well as their family members and friends (especially those who serve as caregivers). For HIV-positive women and MSM, preventable HPV-related diseases and cancers contribute unnecessarily to the burden of diagnosed HIV infection, which is even more concerning given the fact that most of these vulnerable populations are poor and rely on government-funded health insurance plans such as Medicaid, receive healthcare services at Ryan White-funded clinics, and obtain HIV/AIDS-related medications from AIDS drug assistance programs (ADAPs) and pre-exposure prophylaxis programs (PrEP DAPs). Therefore, additional healthcare-related costs for preventable HPV-related diseases and cancers add to the financial burden of an already strained healthcare system, and in our opinion, should be avoided.

Increasing HPV vaccination coverage and adherence to cervical cancer screening guidelines are both *Healthy People 2020* goals for the general population (USDHHS). In this study, we examined HPV-related awareness, knowledge, attitudes, and beliefs among staff at ASOs located in the South U.S. Census region—a geographical area disproportionately affected by HIV/AIDS (5). We further compare our ASO staff to subpopulations of the Health Information National Trends Survey (HINTS) respondents who also resided in the South Atlantic division of the South United States Census region. Our primary rationale for conducting this study was to better understand factors that can increase HPV vaccination coverage and adherence to cervical cancer screening guidelines in the target populations, and more specifically, how ASOs and their staff can play a role in these cancer prevention effort.

## Materials and methods

### Samples

#### ASO staff

In May-June 2016, 30 staff were recruited from three ASOs located in the South US Census region. The principal investigator (LTW) emailed a description of the research study to leaders at four ASOs located in South Carolina (SC). The three ASO leaders that responded were asked to invite eligible staff to participate. Eligibility criteria included being an employee, volunteer, or community partner who provided HIV/sexually transmitted disease (STD) risk-reduction counseling to PLWH or high-risk HIV-negative men (including MSM) or women.

#### HINTS respondents

The HINTS is a population-based mail survey that was used to obtain a subsample of respondents that resided in the same U.S. Census Region (South) and Division (South Atlantic) as our study participants (13). The HINTS is a publicly available dataset that includes awareness, knowledge, and beliefs questions about HPV and the HPV vaccine. Specific to our study, the HINTS 4, Cycle 4 (H4C4) and HINTS 5, Cycle 1 (H5C1) iterations included HPV knowledge questions about anal, cervical, oral, and penile cancers (14, 15). The H4C4 was conducted from August through November 2014, and the H5C1 was conducted from January through May 2017 (14, 15). We chose to compare our sample to H4C4 and H5C1 samples because these were the most recent data that were collected within 1–2 years (2014 and 2017 respectively) of our research study, which was conducted in May/June 2016. Overall response rates for H4C4 and H5C1 were 34.44 and 32.39% (respectively). Published methodological reports are available for details about the HINTS sampling design and data collection methods (14, 15).

### HPV-related measures

Participants in our ASO study completed a 30-min, self-administered, 118-item paper and pencil survey that included questions about HPV and HPV vaccine awareness, knowledge, and attitudes/beliefs drawn heavily from the HINTs survey. For this analysis, we are only focusing on these domains (i.e., awareness, knowledge, and attitudes/beliefs). The principal investigator (LTW) conducted the survey in a group setting and was able to provide oversight to ensure that the respondents did not get help answering the knowledge questions from either from the Internet or their colleagues.

#### Awareness

Both samples were asked if they had ever heard of HPV and the HPV vaccine. We used an adapted version of the HINTS HPV vaccine awareness question to ask our ASO sample if they had heard of specific HPV vaccines (i.e., Cervarix®, Gardasil®, Gardasil-9®) and cancer screening tests (i.e., anal Pap test, HPV test). Response options were yes/no.

#### Knowledge

Both samples were asked about knowledge that HPV can cause anal, cervical, oral, and penile cancers; is a sexually transmitted disease; and will usually clear (i.e., go away on its own without treatment). We used an adapted version of the HPV knowledge question to assess the ASO sample's knowledge that HPV causes more health problems for PLWH and other types of cancers (i.e., vaginal, vulvar). Questions from older HINTS iterations and existing surveys were adapted to assess the ASO sample's HPV vaccine knowledge about the following: main purpose is to prevent HPV infection, 3-doses recommended for females and males (including PLWH) through 26 years old, not recommended for persons older than 26 years old, cervical cancer screening is still needed (13), and safe and effective (16).

#### Attitudes/beliefs

In both samples, beliefs about the HPV vaccine's cervical cancer prevention effectiveness was assessed. We adapted the HINTS HPV vaccine effectiveness question to include other HPV-related cancers (i.e., anal, oral, penile, vaginal, and vulvar). A similar question from an older HINTS iteration was used to assess ASO staff's beliefs that the Pap test was effective at detecting cervical cancer in its earliest stages when treatment is more effective. Response options were: not at all successful, a little successful, pretty successful, very successful, and don't know. We dichotomized this variable as successful or not at all successful/don't know.

We also adapted the HINTS question about cervical cancer being preventable to include other HPV-related cancers (i.e., anal, oral, penile, vaginal, and vulvar). ASO staff who worked with female clients were asked how much they agreed/disagreed that anal, cervical, oral, vaginal, and vulvar cancers were preventable. Similarly, ASO staff who worked with MSM clients were asked if anal, oral, and penile cancers were preventable. Response options were: strongly agree, agree, disagree, strongly disagree: We dichotomized these questions as agree or disagree.

#### Barriers

We used a single item to assess ASO staff's perceptions of barriers that their clients might encounter when trying to access the HPV vaccine, cervical cancer screening tests (i.e., Pap test, HPV test), and/or follow-up treatment or diagnostic tests if their Pap test result was unclear or abnormal. Response options included patient (i.e., childcare), provider (i.e., HIV stigma, HPV stigma), and system-level (i.e., clinic hours, cost, health insurance, transportation, travel distance) barriers. An open-ended response option was used to assess other barriers.

### Data analysis

Stata/SE 14.2 (College Station, Texas, USA) was used to perform all data analyses. We restricted our HINTS data analyses to the subpopulation of US adults who lived in the South Atlantic division of the South US Census division and had no missing observations for any of our covariates (H4C4: *n* = 365/820 South Atlantic division residents; H5C1: *n* = 323/754 South Atlantic division residents). Frequencies and percentages were calculated independently for each of the three study samples. The variability between our research study design and the HINTS sample design and weighting procedures limited our ability to merge datasets. Although we were not able to perform chi-square tests between our sample and each of the HINTS samples, we were able to use proportion tests to determine whether or not the observed differences in HPV-related awareness, knowledge, and beliefs between our sample and the HINTS samples were statistically significant.

## Results

### Sample characteristics

ASO staff were younger (mean age: 47.7 ± 12.5 years; H4C4: 49.5 ± 15.7, *p* = 0.03; H5C1: 50.8 ± 15.3, *p* = 0.0003), 83% non-Hispanic Black or African American (H4C4: 27%, *p* = 0.0036; H5C1: 19%, *p* = 0.0000), and 77% had completed a college degree or higher (77% postgraduate degrees; H4C4: 58%, *p* = 0.0000; H5C1: 62%, *p* = 0.0000) compared to US adults residing in the South Atlantic division of the South U.S. Census region. (Table 1) Forty percent of ASO staff were lesbian or gay. On average, ASO staff had a long tenure at their organizations (8.5 ± 7.1 years), had worked with PLWH for more than a decade (11.4 ± 7.7 years), and served many clients (89.0 ± 85.2 total). Most (76%) were employed full (*n* = 22) or part-time (*n* = 1) at their ASOs, and almost all worked with HIV-positive (or high-risk HIV-negative) women (93%) or MSM (97%) clients (Table 2).

**Table 1:** Characteristics of ASO staff compared to US adults^a^.

**Sample (Data Collection Dates) Sample Size**	**ASO Staff** **(May—June 2016)** ***n* = 30**	**HINTS 4 Cycle 4** **(Aug—Nov 2014)** ***n* = 365**	**HINTS 5 Cycle 1** **(Jan—May 2017)** ***n* = 323**
**Characteristics**		**Mean (SD)**
Age, years	47.7 ± 12.5^b^	49.5 ± 15.7^*^	50.8 ± 15.3^***^
		***n*** **(%)**	
**AGE, YEARS**			
≥50	12 (43)	192 (53)^***^	173 (54)^***^
**Sex**			
Male	16 (53)	116 (32)^***^	108 (33)^***^
**Race/ethnicity**			
Non-Hispanic Black or African American	25 (83)	97 (27)^**^	61 (19)^***^
**Marital status**			
Married	11 (37)	195 (53)^***^	198 (61)^***^
**Education**			
College graduate or more	23 (77)	212 (58)^***^	200 (62)^***^
**ANNUAL HOUSEHOLD INCOME, US DOLLARS ($)**^c^			
≥50,000	12 (40)	224 (61)^***^	212 (66)^***^
**AWARENESS**			
Heard of HPV	30 (100)	365 (100)*^ns^*	323 (100)*^ns^*
Heard of the HPV vaccine	23 (77)	316 (87)^***^	278 (86)^***^
**KNOWLEDGE**			
HPV can cause anal cancer	20 (67)	100 (27)^***^	113 (35)^***^
…cervical cancer	30 (100)	293 (80)^***^	271 (84)^***^
…oral cancer	11 (38)	118 (32)^*^	122 (38)*^ns^*
…penile cancer	17 (59)	110 (30)^***^	126 (39)^***^
…vaginal cancer	25 (83)	–	–
…vulvar cancer	14 (48)	–	–
HPV is a sexually transmitted disease	24 (80)	235 (64)^***^	231 (72)^***^
HPV will usually go away on its own	24 (80)	33 (9)^***^	32 (10)^***^
**BELIEFS**			
HPV vaccine can prevent anal cancer	10 (33)	–	–
…cervical cancer	14 (47)	147 (40)^*^	137 (43)*^ns^*
…oral cancer	11 (48)	–	–
…penile cancer	9 (30)	–	–
…vaginal cancer	11 (48)	–	–
…vulvar cancer	11 (48)	–	–

ASO, community-based HIV/AIDS service organization; HINTS, Health Information National Trends Survey; HPV, human papillomavirus; SD, standard deviation; US, United States).

a Adults located in the South Atlantic division of the South US Census region which includes SC and eight other states (DC, DE, FL, GA, MD, NC, VA, and WV) (17).

b n = 28/30.

c Not sure/refused (n = 3)|

* p < 0.05;

** p < 0.01;

*** p < 0.001.

ns *p > 0.05*.

**Table 2:** Other characteristics of ASO staff (*n* = 30).

	**Mean (SD)**
**CHARACTERISTICS**	
#Years worked with PLWH	11.4 ± 7.7
#Years worked at ASO (*n* = 28)	8.5 ± 7.1
#Clients served, total (*n* = 25)	89.0 ± 85.2
	***n*** **(%)**
**SEXUAL ORIENTATION, SEX**	
Heterosexual, female	12 (40)
Heterosexual, male	6 (20)
Lesbian or gay	12 (40)
Employed full time at ASO	23 (76)
Worked with HIV-positive women (or HIV-negative women engaging in HIV risk behaviors)^a^	28 (93)
Worked with MSM (HIV-positive and HIV-negative)	29 (97)
Worked with clients ≤ 26 years old	23 (100)^b^
Clients have talked to them about the HPV vaccine	7 (30)^b^
Worked with clients who had initiated the 3-dose HPV vaccine series	7 (30)^b^
Worked with clients who had completed the 3-dose HPV vaccine series	4 (17)^b^
Worked with clients who had been diagnosed with an HPV-related disease or cancer	18 (60)
**AWARENESS**	
Heard of Cervarix®	12 (52)^b^
… Gardasil®	8 (35)^b^
… Gardasil-9®	18 (78)^b^
Heard of the HPV test	24 (80)
Heard of an anal Pap test	19 (66)
**KNOWLEDGE**	
HPV causes more health problems for PLWH	24 (80)
Main purpose of the HPV vaccine is to prevent HPV infection	19 (83)^b^
Three doses of the HPV vaccine are recommended^c^	9 (39)^b^
HPV vaccine is recommended for females and males ≤ 26 years old	19 (83)^b^
HPV vaccine is recommended for PLWH 9-26 years old	16 (70)^b^
HPV vaccine is not recommended for people >26 years old	6 (26)^b^
Females who have gotten the HPV vaccine still need to get screened for cervical cancer	21 (91)^b^
HPV vaccine is safe	12 (52)^b^
HPV vaccine is effective	10 (43)^b^
**BELIEFS**	
Pap test can detect cervical cancer in its earliest stages	26 (87)
There's not much a woman can do to lower her chances of getting anal cancer	1 (3)
… cervical cancer	1 (3)
… oral cancer	2 (7)
… vaginal cancer	13 (43)
… vulvar cancer	1 (3)
There's not much a man can do to lower his chances of getting anal cancer	1 (3)
… oral cancer	1 (3)
… penile cancer	13 (45)
**HEALTH CARE ACCESS BARRIERS**	
**PATIENT-LEVEL**	
Childcare	8 (27)
**INTERPERSONAL**	
HPV stigma	19 (63)
HIV stigma	8 (27)
**SYSTEM-LEVEL**	
Clinic hours	23 (77)
Cost	18 (60)
Health insurance	15 (50)
Transportation	15 (50)
Travel distance	14 (47)
Other	10 (33)

ASO, community-based HIV/AIDS service organization; HIV, human immunodeficiency virus; HPV, human papillomavirus; SD, standard deviation.

a For example, diagnosed with a sexually transmitted disease within the past year.

b n = 23 / 30 who had heard of the HPV vaccine.

c *3-dose HPV vaccine series was the only recommendation at this time (and is the current recommended for people living with HIV (18)*.

### HPV-related awareness, knowledge and attitudes/beliefs

#### HPV

In the ASO staff and both HINTS samples, all had heard of HPV. Compared to US adults residing in the South Atlantic division, all ASO staff knew that HPV can cause cervical cancer (H4C4: 80%, *p* = 0.0000; H5C1: 84%, *p* = 0.0000). A larger percentage of ASO staff knew that HPV can cause anal (67%), penile (59%), and oral (38%) cancers compared to US adults residing in the South Atlantic division: anal (H4C4: 27%, *p* = 0.0000; H5C1: 35%, *p* < 0.05); oral (H4C4: 32%, *p* = 0.0236; H5C1: 38%, *p* = 0.9324); and penile (H4C4: 30%, *p* = 0.0000; H5C1: 39%, *p* = 0.0000), and cancers. Eighty-three percent of ASO staff knew that HPV can cause vaginal cancer, and almost half (48%) knew HPV can cause vulvar cancer. The proportions of ASO staff who knew that HPV is an STD (80%) that will usually go away on its own without medical treatment (or clear; 80%) were higher compared to US adults residing in the South Atlantic region: HPV is an STD (H4C4: 67%, *p* = 0.0000; H5C1: 74%, *p* = 0.0001), …that will usually clear (H4C4: 10%, *p* = 0.0000; H5C1: 9%, *p* = 0.0000; Table 1) Most ASO staff (80%) knew that HPV causes more health problems for PLWH (Table 2).

#### HPV vaccine

A smaller percentage (77%) of ASO staff (*n* = 23/30) had heard of the HPV vaccine (Table 1), (which included 78% (*n* = 18/23) who had heard of the nonavalent HPV vaccine (i.e., Gardasil-9®; Table 2), compared to 87% (H4C4: *p* = 0.0000) and 86% (H5C1: *p* = 0.0001) of US adults residing in the South Atlantic division (respectively; Table 1). Although 70% of ASO staff knew that the HPV vaccine was recommended for PLWH aged 9–26 years, only 52 and 43% (respectively) thought the HPV vaccine was safe and effective.

Almost all ASO staff (91%) knew that vaccinated females still needed to be screened for cervical cancer. The HINTS 3 (2008) iteration is the last time that this survey question was assessed. Comparatively 94% of US adults at that time knew that vaccinated females still needed to be screened for cervical cancer (*p* = 0.59; data not shown). While 3–7% of ASO staff thought that anal, cervical, oral, and vulvar cancers were not preventable, almost half thought that penile (45%) and vaginal (43%) cancers were not preventable (Table 2).

Compared to US adults residing in the South Atlantic region (H4C4: 85%; H5C1: 83%), 77% of ASO staff had heard of the HPV vaccine. Almost half (47%) of ASO staff thought that the HPV vaccine can prevent cervical cancer, which was a statistically significant higher than the proportion of US adults residing in the South Atlantic region (H4C4: 40%, *p* = 0.0100) in 2014. However, compared to US adults residing in the South Atlantic region in 2017, the proportion of ASO staff (47%) that thought the HPV vaccine can prevent cervical cancer was not statistically higher (H5C1: 43%, *p* = 0.0987). Fewer than half of ASO staff thought the HPV vaccine could prevent anal (33%), oral (48%), penile (30%), vaginal (48%), and vulvar (48%) cancers (Table 1).

**Figure 1:**
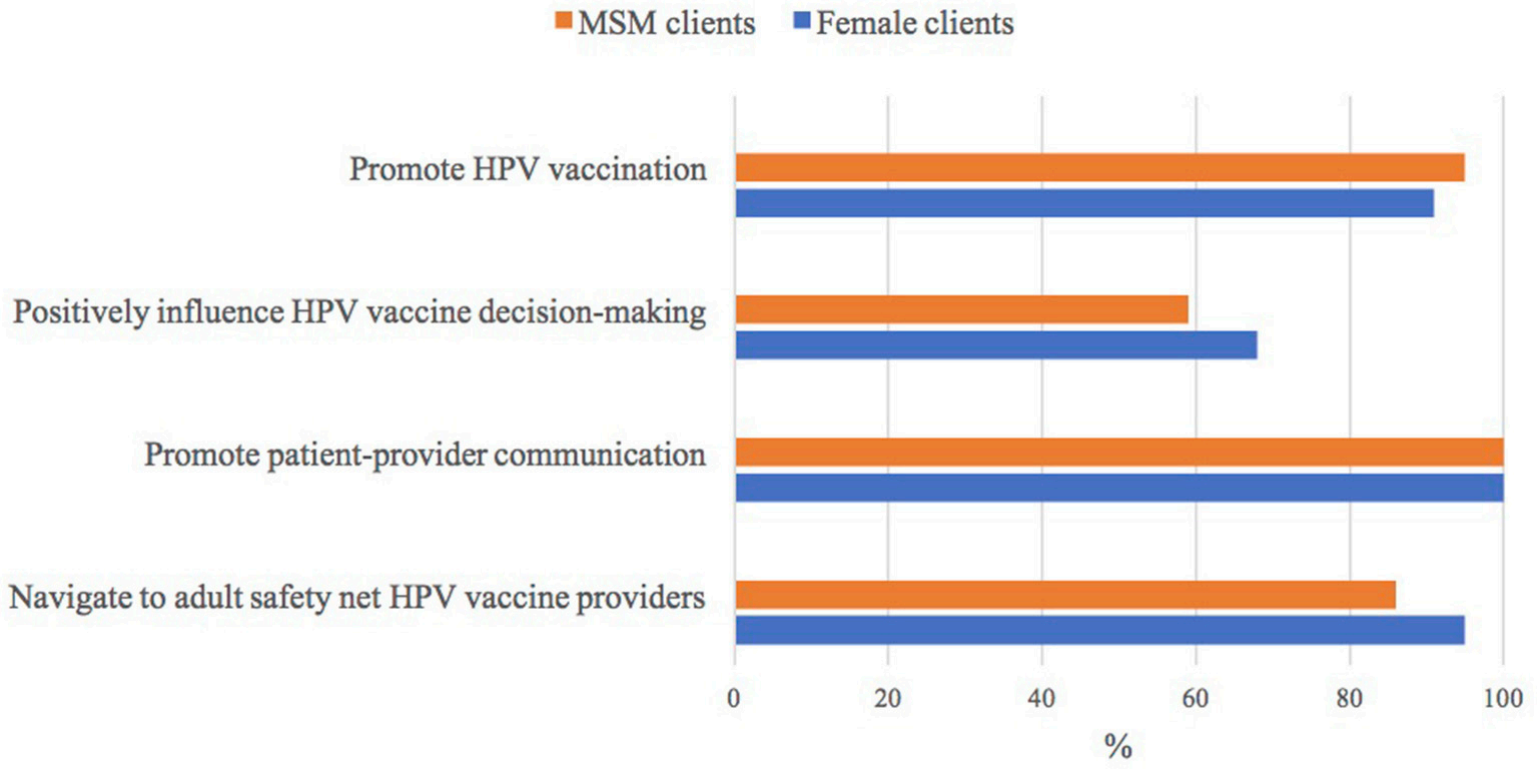
ASO staff's attitudes toward human papillomavirus (HPV) vaccine | Notes: *n* = 22/23 who had heard of the HPV vaccine; ASO (community-based HIV/AIDS service organization); MSM (men who have sex with men).

All ASO staff were willing to encourage their female and MSM clients to talk to their healthcare providers about the HPV vaccine. A larger percentage of ASO staff who worked with MSM clients (95%) were willing to encourage these males to get the HPV vaccine, compared to ASO staff who worked with female clients (91%; *p* = 0.47). In contrast, a smaller proportion of ASO staff who worked with MSM clients thought their opinion could influence these male's HPV vaccine decision-making (59%) than ASO staff who worked with female clients (68%; *p* = 0.37). Similarly, a smaller proportion of ASO staff who worked with MSM clients were willing to help navigate these males to adult safety net HPV vaccine providers (86%) where they could get the HPV vaccine for free or at a reduced cost than those who worked with female clients (95%; *p* = 0.06; Figure 1).

#### Cancer screening

Eighty-seven percent of ASO staff thought the Pap test was effective at detecting cervical cancer in its earliest stages. (Table 2) Comparatively, the last time this HINTS question was assessed in 2012, fewer US adults (61%) thought that the Pap test was an effective cervical cancer screening tool (data not shown).

All ASO staff who worked with female clients were willing to ask these females when did they had their most recent Pap test, encourage unscreened/underscreened females to talk to their health care provider about getting a Pap test, and if applicable, also encourage these females to follow up with their health care provider if abnormal follow-up care was needed. Almost all ASO staff who worked with female clients were willing to ask them if their most recent Pap test result was normal, unclear, or abnormal (96%), and were also willing to help these females better understand their Pap test results (93%; Table 3).

**Table 3:** Attitudes towards cervical cancer screening among 28 ASO staff who worked with HIV-positive (or at-risk HIV-negative) female clients.

**CHARACTERISTICS**	
	*n* (%)
**CERVICAL CANCER SCREENING**	
Pap test	
Willing to ask female clients, *How long ago did you have your most recent Pap test?*	28 (100)
*Was your most recent Pap test result normal, unclear, or abnormal?*	27 (96)
Willing to encourage unscreened/underscreened female clients to talk to their health care provider about getting a Pap test	28 (100)
Willing to help female clients better understand their Pap test results	26 (93)
Willing to encourage females clients to follow up with their health care provider if abnormal follow-up care was needed	28 (100)
**HPV DNA TEST**	
Willing to encourage their female clients to talk to their health care provider about getting an HPV test if applicable	28 (100)
**HEALTH CARE ACCESS BARRIERS**	
Willing to help female clients find free or low-cost cervical cancer screening tests (i.e., Pap test, HPV DNA test)	27 (96)

*ASO, community-based HIV/AIDS service organization*.

Most ASO staff had heard of the HPV DNA test (80%; Table 2), and all who worked with female clients (*N* = 28) were willing to encourage females aged 30+ years to talk to their healthcare provider about getting an HPV DNA test. Almost all ASO staff (96%; *N* = 27/28) that worked with female clients were willing to help these females find free or low-cost cervical cancer screening tests (i.e., Pap test, HPV DNA test; Table 3).

**Figure 2:**
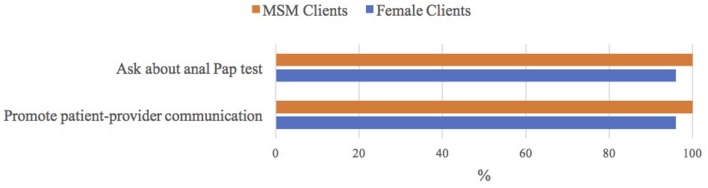
ASO staff's attitudes toward anal Pap test|Notes: *n* = 29/30 worked with men who have sex with men (MSM) clients; *n* = 28/30 worked with female clients; ASO (community-based HIV/AIDS service organization).

Many ASO staff had heard of the anal Pap test (66%; Table 2). All ASO staff who worked with MSM clients were willing to talk to these males about the anal Pap test and encourage them to talk to their health care provider about getting a Pap test, compared to 96% of ASO staff who worked with female clients (*p* = 0.26; Figure 2).

#### Perceived barriers to HPV-related cancer prevention tools

Only 27% of ASO staff identified childcare as a barrier for their clients. While 63% thought HPV stigma was a barrier, only 27% thought HIV stigma was a barrier for their clients. Beyond HPV stigma, most of the barriers were related to healthcare access and included the following: 77% clinic hours, 60% cost, 50% health insurance, 50% transportation, 47% travel distance. An open-ended response options was included to capture other barriers, of which 33% of ASO staff identified: “*confidentiality”; “access to the vaccine”;* “*knowledge, education, and understanding”; “lack of knowledge about it and the importance of it”; “lack or limited knowledge of HPV and its impact on health”; client not able to full understand the material provided. Make it as clear as possible-6 grade level”; “shared information – brochures, pamphlets”; “fear of the disease and trust of the health system. Tuskegee Study Institute”; “side effects of medication / caregivers”* (Table 2).

## Discussion

Community-based ASOs provide PLWH, MSM, and other vulnerable populations with valuable resources such as health education, patient navigation, and social support. Some of the ASO staff that we recruited for our research study had high levels of awareness and knowledge about HPV and some effective HPV-related cancer prevention (i.e., HPV vaccination) and screening (i.e., HPV test) tools. This may be attributed to the disproportionate burden of HPV-related diseases and cancers among their clients. That said, the lower level of awareness about the anal Pap test raised some concern given the high proportion of ASO staff that participated in our research study who work with MSM clients and the high burden of anal diseases and cancers among MSM (which is further exacerbated by HIV infection). ASO staff's lack of knowledge about anal Pap tests could be attributed (at least in part) to the fact that in contrast to cervical cancer, there are no recommended screening guidelines for anal cancer. However, the general sense is that for high-risk populations such as MSM (and especially HIV-positive MSM), the potential for improving HPV-related cancer outcomes outweigh the potential harm. In this regard, the potential for ASO staff to help their clients overcome barriers and enable them to make informed (or at a minimum shared) decisions about cancer screening with their health care providers becomes increasingly important (19).

Although some ASO staff may not share the same positive attitudes and beliefs about HPV vaccination and anal and/or cervical cancer screening, almost all of the ASO staff in our research study were willing to promote patient-provider communication and provide patient navigation support to their clients. Studies have shown that ASO clients' health outcomes are positively associated with how effectively ASOs can integrate various services (20). Further research is needed to better understand how to integrate HPV-related cancer prevention education into existing services provided by ASO staff, which include HIV/STD testing and risk-reduction counseling for both PLWH and high-risk HIV-negative persons. Because many ASO staff also help their clients link to healthcare providers where they can receive needed healthcare services such as HIV care and pre-exposure prophylaxis (PrEP) clinics, the integration of patient navigation to adult HPV vaccination safety net providers and other cancer care providers where they can receive screening, diagnostic, and treatment services also warrant further study. ASOs have a track record of success in helping their clients overcome healthcare access barriers. Further, ASO staff that participated in our research study were willing to become engaged in HPV-related cancer prevention efforts. In this regard, failure to leverage ASO staff's ability to reach marginalized, high cancer risk populations such as PLWH and MSM is a missed opportunity that warrant further study at least.

### Strengths

This study is one of only a few that have compared the US population-based HINTS sample to a special subpopulation of US adults (21, 22). Our sample was a predominantly minority group whom the majority were college graduates compared to the HINTS samples, which were both a predominantly white group whom only about half were college graduates. This study is among a few that have compared their study samples' HPV-related awareness, knowledge, attitudes/beliefs to a HINTS sample (21, 22). We feel this is a missed opportunity to further our understanding of how to promote the prevention and control of HPV-related diseases and cancers beyond population-based samples like HINTS to include special populations such as PLWH and MSM, or even less understudied populations such as ASO staff. Another strength of our study (in contrast to a population-based study like HINTS) is that we were able to ask more specific questions about HPV-related awareness (e.g., anal Pap, HPV test), knowledge (HPV can cause vaginal and vulvar cancers), and beliefs (HPV vaccine can prevent HPV-related cancers other than cervical cancer), as compared to the HINTS questions that were developed for the general population.

### Limitations

The main limitation of our study is its small sample size. However, it should be noted that our study was a sample of provider-level participants, and not a population-level sample. Further, our sample of provider-level participants served a vulnerable subgroup of the population (i.e., PLWH, MSM, and other vulnerable populations). Although we were able to ask more specific questions, this limited our ability to make additional comparisons between our sample and the HINTS sample. Our study findings may not be generalizable to other ASO staff in other locales. Another limitation is that we did not assess gay stigma as a potential healthcare access barrier.

## Conclusion

### Implications for future research and practice

HPV vaccination can reduce the burden of HPV vaccine preventable diseases and cancers. Future research to assess what ASO staff's beliefs are about the HPV vaccine specifically for special populations such as PLWH and MSM is warranted, especially since most knew that the HPV vaccine was recommended for PLWH, but only about half (or fewer) thought that the HPV vaccine was safe and effective. Similarly, early detection of precancerous cells can improve health outcomes. Because ASO play an important role in improving health outcomes for their clients (23), it seems logical that engaging their staff in HPV-related cancer prevention efforts may be a viable approach to increasing HPV vaccination rates and cervical cancer screening adherence among PLWH, MSM, and other high-risk vulnerable populations. Many HIV-positive women [including those with low health literacy; (24)] have abnormal Pap test histories (25) and thus ASO staff's willingness to help HIV-positive women understand their Pap test results and encourage them to follow-up with their healthcare providers could play a vital role in reducing cervical cancer disparities among HIV-positive women.

## Ethics statement

Data collection activities for this research study were carried out in accordance with the recommendations of the University of South Carolina's Office of Research Compliance with verbal informed consent from all subjects that participated in our needs assessment survey. All participants gave verbal informed consent in accordance with the Declaration of Helsinki. The protocol was approved by the institutional review board of the University of South Carolina's Office of Research Compliance.

## Author contributions

LW conceptualized the research project and HB (along with LW's other K01 mentors contributed to the study design). LW conducted the literature review that informed the development of the survey, which LW developed and HB and SB contributed to expert review and content validity. LW conducted the research study (which included all aspects of data acquisition). LW lead data analysis with assistance from NS. All authors (LW, SB, NS, HB, and MO) have all contributed to the interpretation of the findings. LW provided the initial draft of the findings, and all authors (LW, SB, NS, HB, and MO) assisted with revising subsequent drafts for important intellectual content. All authors (LW, SB, NS, HB, and MO) read and provided approval for publication of the final draft of the manuscript that was submitted. LW along with her K01 mentors (HB and MO) agreed to be accountable for all aspects of the work in ensuring that questions related to the accuracy or integrity of any part of the work were appropriately investigated and resolved.

### Conflict of interest statement

HB was and still is a member of the United States HPV Advisory Board of Merck. The remaining authors declare that the research was conducted in the absence of any commercial or financial relationships that could be construed as a potential conflict of interest.
